# Deep learning-based multi-model approach on electron microscopy image of renal biopsy classification

**DOI:** 10.1186/s12882-023-03182-6

**Published:** 2023-05-09

**Authors:** Jingyuan Zhang, Aihua Zhang

**Affiliations:** 1 Children’s Hospital of Nanjing Medical University, 72 Guangzhou Road, Nanjing, China; 2 Laboratory Medicine Center, The Second Affiliated Hospital of Nanjing Medical University, Nanjing, 210011 China

**Keywords:** Biopsy, Deep Learning, Diagnostic Imaging, Model, Renal

## Abstract

**Background:**

Electron microscopy is important in the diagnosis of renal disease. For immune-mediated renal disease diagnosis, whether the electron-dense granule is present in the electron microscope image is of vital importance. Deep learning methods perform well at feature extraction and assessment of histologic images. However, few studies on deep learning methods for electron microscopy images of renal biopsy have been published. This study aimed to develop a deep learning-based multi-model to automatically detect whether the electron-dense granule is present in the TEM image of renal biopsy, and then help diagnose immune-mediated renal disease.

**Methods:**

Three deep learning models are trained to classify whether the electron-dense granule is present using 910 electron microscopy images of renal biopsies. We proposed two novel methods to improve the model accuracy. One model uses the pre-trained ResNet convolutional layers for feature extraction with transfer learning which was firstly improved with skip architecture, then uses Support Vector Machine as the classifier. We developed a multi-model to combine the traditional ResNet model with the improved one to further improve the accuracy.

**Results:**

Deep learning-based multi-model has the highest model accuracy, and the average accuracy is about 88%. The improved ReseNet + SVM model performance is much better than the traditional ResNet model. The average accuracy of the improved ResNet + SVM model is 83%, while the traditional ResNet model accuracy is only 58%.

**Conclusions:**

This study presents the first models for electron microscopy image classification of Renal Biopsy. Identifying whether the electron-dense granule is present plays an important role in the diagnosis of immune complex nephropathy. This study made it possible for Artificial Intelligence models assist to analyze complex electron microscopy images for disease diagnosis.

## Background

Renal disease is very common, and approximately 750 million people in the world are suffering from it [1]. Renal biopsy is the gold standard for diagnosing renal disease [2]. Besides, Transmission Electron Microscope (TEM) is performed routinely on renal biopsies for its value in pathomorphological diagnosis, as it can examine the ultra-structure [3, 4]. Especially for immune-mediated renal disease diagnosis, whether the electron-dense granules present or not in the electron microscope report are of vital importance. However, electron microscope images on renal biopsy are currently assessed by pathologists with visual estimation, and it usually takes a long time to get the electron microscope results, which caused the problem of electron microscopy reports to lag far behind the need for clinicians to shorten the Turnaround Time [5] and get electron microscope reports as soon as possible. What’s more, the electron microscope requires experienced pathologists to perform. Different pathologists have different diagnostic standards and levels. Since different pathologists have different working years and professional titles, the final judgment results may not be the same. Electron Microscopy is expensive to perform. Especially when conducting scientific research, for example, it is necessary to build an animal model to check the effect of the knockout gene on lupus nephritis, and the electron microscope is necessary to assess whether the lupus nephritis animal model is successfully constructed or not [6]. All of the above reasons limit the widespread use of electron microscopes.

Deep learning algorithms characterized by Convolutional Neural Networks (CNN) can greatly improve the performance of many visual classification problems. However, a few studies of deep learning models on renal biopsy are reported worldwide. Meyke hermsen et al. [7] recently developed a CNN-based model for multiclass segmentation of renal tissue stained by periodic acid-Schiff. Brandon Ginley et al. [8] proposed an RNN-based pipeline to classify biopsy samples from 54 patients with diabetic nephropathy. To our knowledge, this is the first work to train deep learning models on a TEM image of renal biopsy. Since the TEM image of renal biopsy is greyscale and the PAS image is colorful, the morphological changes in the greyscale image (TEM) are much harder to identify for the computer [9]. What’s more, typically in most research, it is one model handling all visual feature conditions. Although a single model may be accurate on average, there is still a big chance that a single model will miss some important features due to the model uncertainty [10]. There’s almost no public research on deep learning models on TEM images of renal biopsy. Also, in the pathological image classification field, few multi-model research is addressed.

The goal of this study was to develop and test a novel method that combines improved deep learning models with multi-model to automatically classify whether the electron-dense granule is present in the TEM image of renal biopsy. To assist the pathologists to diagnose immune-mediated renal disease more quickly and easily, whether the electron-dense granules present in the electron microscope image will be classified by the deep learning models and multi-model. In this novel method: firstly two single deep learning models were developed and trained. One single deep learning model is the conventional ResNet Model. Another single model uses the improved ResNet convolutional layers with skip architecture which was firstly proposed in this paper, then uses the classical machine learning method Support Vector Machine (SVM) as the classifier. Finally, to improve the classification accuracy, the multi-model will combine the two trained deep learning models into one model with Artificial Neural Network algorithms. All three models are trained for classifying whether the electron-dense granules are present in the TEM image or not.

## Methods

### Renal biopsy samples description

The 910 images are from 319 renal biopsies during the period from August 2017 to June 2019. The renal needle-core biopsies in this paper are obtained from 319 patients from Children’s Hospital of Nanjing Medical University in China. All of the patients are children whose ages are younger than 18.

We select 319 patients from 1252 patients based on the following standard. Firstly, we choose the specimens from patients who have been clearly diagnosed as immune-mediated renal disease, combined with the results of renal biopsy and clinical symptoms. Secondly, among the electron microscope images of these patients, we select the clear images in which meaningful parts are taken, such as the electron microscope images that preferably contain glomeruli, and electron-dense granule is present in the TEM images. These images are labeled as “positive”. Thirdly, we choose the specimens from patients who are not diagnosed with immune-mediated renal disease, combined with the results of renal biopsy and clinical symptoms. And the electron-dense granule is not present in the TEM images. These images are labeled as “Negative”. According to this standard, 319 renal biopsy specimens were selected from 1252 patients.

The preparation of renal biopsy specimens was done according to international standards [11]. We got the image under the 2 μm ruler.

The above-mentioned TEM images are diagnosed by two experienced renal pathologists. One of the pathologists from the General Hospital of Eastern Theater Command gave the result, and then the other pathologist from the Children’s Hospital of Nanjing Medical University reviewed the result again. If the results of the two pathologists disagreed, our final solution was not to use this sample. In this study, the total number of TEM images with labels is 910. Of the 910 TEM images, 455 images are labeled as “Positive” which means that Electron dense granule is present in the TEM image, and the rest of 455 images are labeled as “Negative” which means Electron dense granules are not present in the TEM image.

All of the renal biopsy samples used in this study have passed the ethical review. And the Approval number is 202008074–1.

### Deep learning-based multi-model architecture

This novel algorithm is mainly about developing a multi-model that combines two single deep learning models into one model to further improve classification accuracy. The first single model is the improved ResNet + SVM model, and the second single model is the conventional ResNet model.

### Conventional ResNet50

The ResNet residual component has two types of blocks. One is Identity Block, and the other is Convolutional Block.

1 $$\mathrm{The Identity Block is defined as H}\left(\mathrm{x}\right) =\mathrm{F}\left(\mathrm{x},\left\{\mathrm{Wi}\right\}\right) +\mathrm{x}.$$

where x and H(x) are the input and output vectors of the layers. F(x, {Wi}) is the residual mapping function. {Wi} represents the convolutional layer weights. x is added to the result of F by the shortcut connection path.

**Fig. 1:**
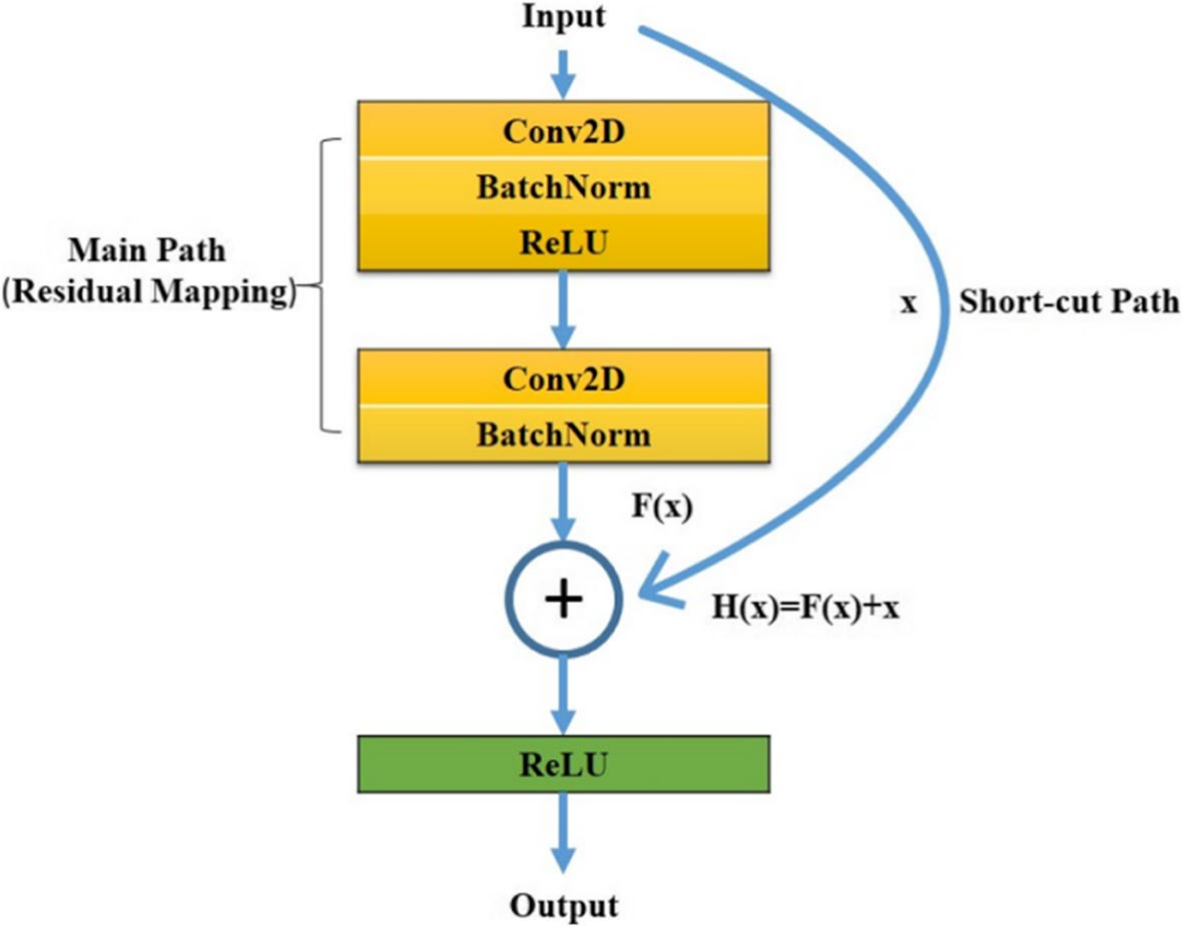
Identity Block Structure

Figure 1 illustrates the structure and detailed process of the Identity Block. Firstly, x is the input vector, and x has two paths to go. One is the main path where x will be transformed by the residual mapping function F. In the main path, F(x,{Wi}) is performed. The main path usually consists of two typical weight layers. One weight layer is composed of a 2D convolutional layer and a Batch-Norm layer which will conduct the Normalization of the channel axis. After the input x multiplied by the weights {Wi} in the first layer, the nonlinear activation function ‘ReLU’ will be used. And then the result will be into the next weight layer. At the same time, the input x will be fed into the other path called the shortcut path. In the shortcut connection, x will be directly added to the result from the main path. Finally, the combined result H(x) will be applied to the ReLU activation function.

2 $$\mathrm{The Convolutional Block calculates H}\left(\mathrm{x}\right) =\mathrm{F}\left(\mathrm{x}, \left\{\mathrm{Wi}\right\}\right) +{\mathrm{W}}_{\mathrm{s}}\mathrm{x}.$$

where x and H(x) are the input and output vectors of the layers. F(x, {Wi}) is the residual mapping function. Different from Identity Block, in the shortcut path, the input x will also be transformed by the 2D convolutional layer in the Convolutional Block. Ws denotes the weights of the convolutional layer in the shortcut path.

**Fig. 2:**
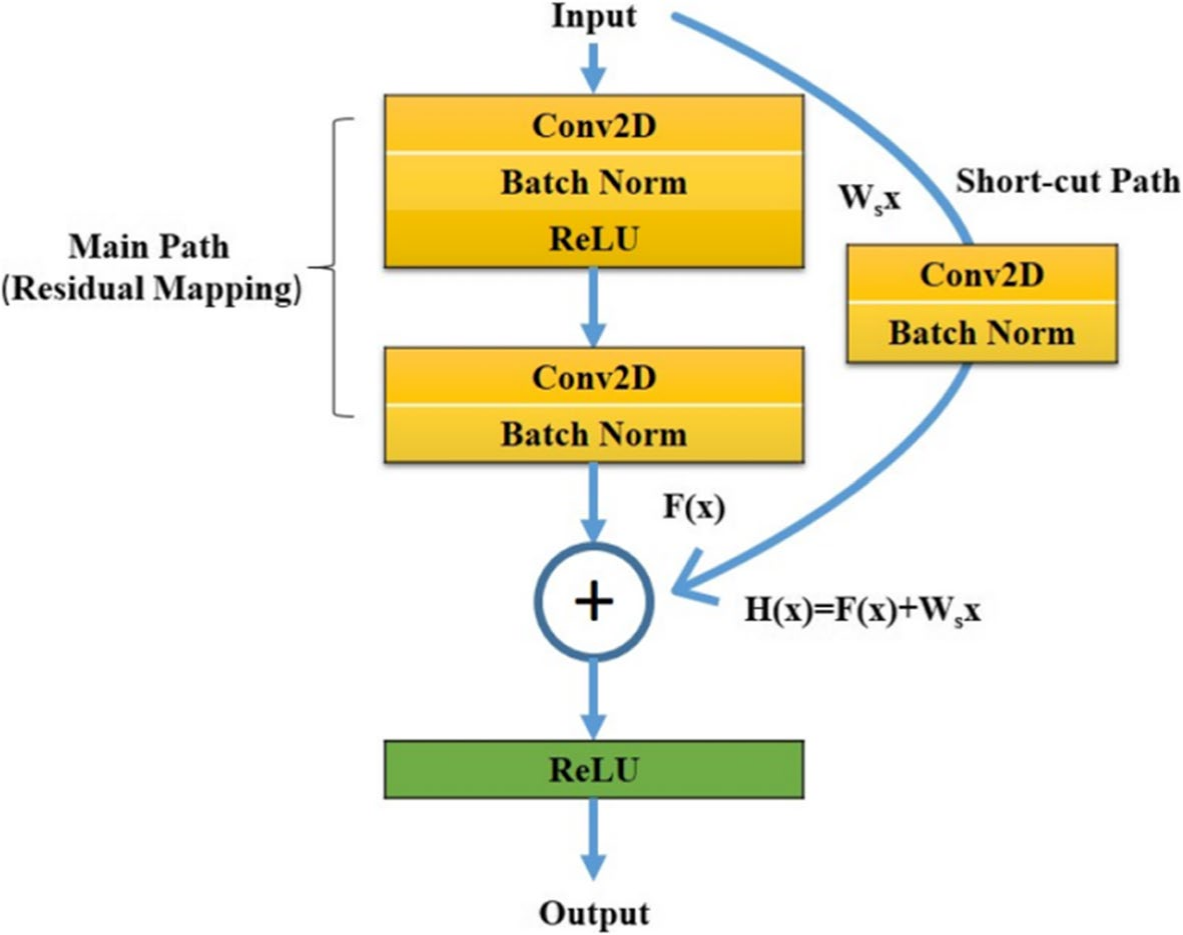
Convolutional Block Structure

Figure 2 shows the structure and process of the Convolutional Block. The only difference from the Fig. 1 is the shortcut path. In Identity Block, the input x is directly added to the result of the main path. However, the input x will be fed into a weight layer then the result is combined with the main path.

### Improved ResNet + SVM model with transfer learning

In this paper, the conventional ResNet model is improved by adding a skip architecture and replaced with an SVM classifier.

ResNet model is the state-of-the-art image classification model. The deeper layer can increase the CNN model accuracy, but it brings a gradient vanishing problem [12]. ResNet model could solve the gradient vanishing problem because of residual network design [13]. As a result, the residual component and very deep layer of ResNet could ensure that the model can extract sufficient image features.

#### Improved Resnet with skip architecture

**Fig. 3:**
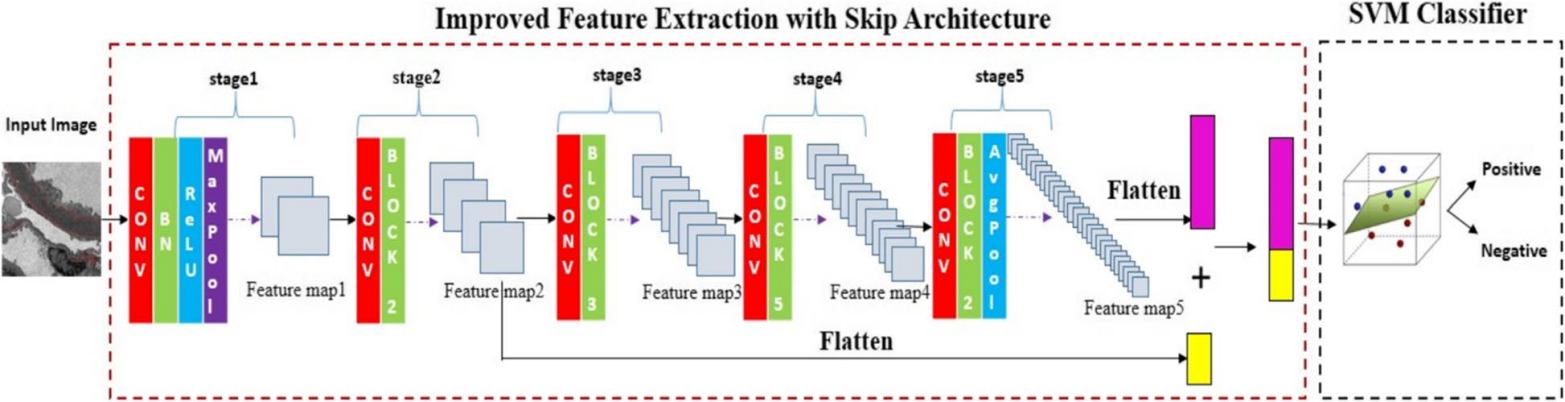
The structure of the Improved ResNet + SVM Model: In the feature extraction part, it combines fine information and coarse information. In the classification part, an SVM classifier is used

As our task is the histopathologic classification of TEM images, it has higher requirements for feature extraction function compared with traditional image classification problems. We improved the feature extraction structure of the ResNet model. As addressed in [14], the deeper layer net tends to extract the global information of an image, since its receptive field is large. Whereas the features extracted by the shallow layer net are partial information of an image, and it focuses on more detailed geometry information of the partial area of an image. In the conventional ResNet50 feature extraction structure, there are about 49 convolutional layers in sequence, so the final extracted features are from the very deep layer. As a result, the final features contain more global and coarse information about the image. However, in our task of classifying electron-dense granules in the TEM image, pathologists pay much attention to partial area information of TEM image by their visual estimations. This requires the extracted features can also contain fine information about the partial area. We developed the skip architecture to combine the deep, coarse layer information with shallow, fine layer information. Figure 3 shows the improved model structure.

1. Firstly, the input image will be resized to 224 × 224x3, and then passed through the stage1. Stage 1 is composed of a 2D Convolution which has 64 kernels with a shape size of (7 × 7) and stride of (2,2), a Batch-Normalization layer, ReLU activation function, and a MaxPooling with the kernel size of (3X3) and stride of (2,2).
2. Secondly, the result from stage 1 will pass through stage2. Stage2 consists of a Convolutional Block and two Identity Blocks. For each block, 3 convolution layers are stacked one over the other where the first layer has 64 filters with a kernel size of (1 × 1), the second layer has 64 filters with a kernel size of (3 × 3), and the last layer has 256 filters with a kernel size of (1 × 1). Since the features extracted by stage2 contain fine and partial information about the image, the output of the stage2 will have two paths, the first path is into stage 3 and pass-through stage 4 and stage 5 in sequence, and the second path will be combined with the final features from stage5 which contain coarse and global information of the image.
3. Thirdly, for stage 3, stage 4, and stage 5, the model structure is similar. Stage 3 has one Convolutional Block and three Identity Blocks. Stage 4 has one Convolutional Block and five Identity Blocks. Stage 5 has one Convolutional Block and two Identity Blocks. Each block has 3 convolution layers stacked. The filter size of the three layers is (1 × 1), (3 × 3), (1 × 1) respectively. For stage 3, stage 4 and stage 5, the filters are corresponding [128,128,512], [256, 256, 1024] and [512, 512, 2048].
4. Finally, the features from stage 5 and stage2 will be concatenated. As a result, the concatenated features will contain global information as well as fine information of the image and then flatten to one dimension.

#### Improved ResNet 50 combined with SVM model

In the improved ResNet 50 model, the Feature extraction component has been changed to get more accurate features. However, in the classification component of the conventional ResNet model, one fully connected layer with the “softmax” function is used for classification. Support Vector Machine is a classical two-binary classifier. Our task is two binary classification problems, so we will try to combine the improved Resnet50 feature extraction part with the SVM classifier.

Support Vector Machine (SVM): The goal of SVM is to find an optimal hyperplane that best separates two-class datasets so that distance from the nearest data points in space is maximized. This non-linear optimization problem can be transformed into a dual problem by the Lagrange method.

$$\mathrm{Objective function}: Max\mathrm{W}\left(\mathrm{\alpha }\right)=\sum_{i=1}^{l}{\alpha }_{i}-\frac{1}{2}\sum_{i,j}^{l}{\alpha }_{i}{\alpha }_{j}{y}_{i}{y}_{j}<\varphi \left({x}_{i}\right)\bullet \varphi \left(x\right)>$$

$$=\sum_{i=1}^{l}{\alpha }_{i}-\frac{1}{2}\sum_{i,j}^{l}{\alpha }_{i}{\alpha }_{j}{y}_{i}{y}_{j}K({x}_{i},x)$$

3 $$\mathrm{s}.\mathrm{t }\sum_{i=1}^{l}{\alpha }_{i}{y}_{i}=0, 0\le {\alpha }_{i}\le C;i=\mathrm{1,2},\dots ,l$$

where $\mathrm{\alpha }$ is the Lagrange multiplier, $\mathrm{y}$ is the support vector, C is the penalty factor, $<\varphi \left({x}_{i}\right)\bullet \varphi \left(x\right)>$ is the nonlinear kernel function, and x is the input dataset.

### Multi-model

Although deep learning models can greatly improve prediction accuracy, they still have errors. Model errors are affected by many uncertainties from various sources, such as the observation data noise, and model structural deficiencies [15]. Recently, some works about model uncertainty in deep learning have been published [16–18]. One way to reduce the model uncertainty is a multi-model approach [19]. The multi-model approach is to combine predictions from multiple models. This idea was explored more than 40 years ago with some studies in econometrics and statistics [20–23].

Ajami et al. [24] proposed a new scheme that seeks to obtain a consensus from a combination of multiple model predictions, so that one model’s output errors can be compensated by others’ in hydrological model prediction. One of the combination techniques is to use the deterministic weights to combine multiple model outputs [25]. The weighting strategy typically tries to give higher weights to the better-performing models. This approach can produce consensus predictions that are better than those from a single model [26, 27].

However, in the computer vision field, to our knowledge, this is the first work to develop the multi-model scheme with ANN weighting strategy to further improve the deep learning models' accuracy.

### ANN Based multi-model scheme

The simulated output from the individual model mostly has a nonlinear relationship with the ground truth. While Neural Network Method can model complex non-linear relationships, particularly in situations where the explicit form of the relation between the variables involved is unknown. The NNM can integrate information from physically different sources.

**Fig. 4:**
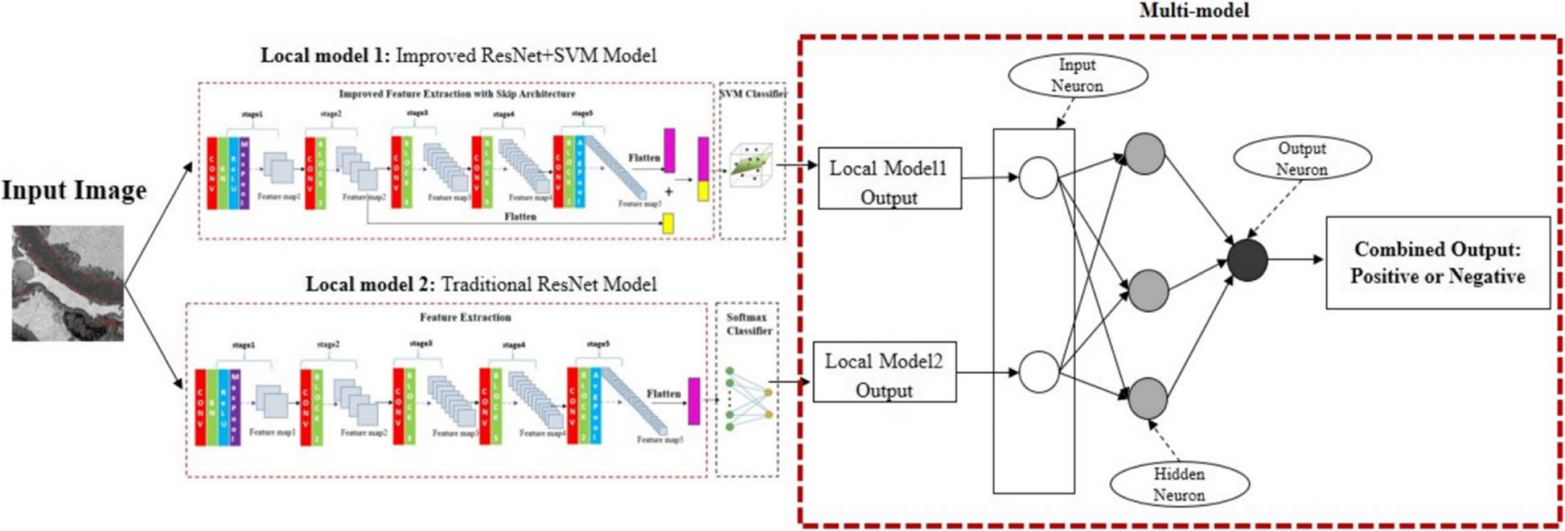
Deep Learning-Based Multi-model Approach Architecture

Figure 4 shows the ANN Based Multi-model algorithm scheme. The original input image will be fed into two models respectively. One model is the improved ResNet + SVM model, and the other is the conventional model. The input layer has two neurons, the first input is the prediction from the improved ResNet + SVM model, and the second input neuron is output from the conventional ResNet model. There is one hidden layer and an output layer. In the output layer, the neuron means the ground-truth label.

### Dataset for model

We divided 910 images into a developing cohort and a validation cohort in this study. We divided 910 images into two cohorts. The developing cohort for the training model, and the validation cohort for calculating and comparing the model accuracy. The 910 images are divided into three parts: one part contains 374 images, another part contains 352 images, and the other part contains 184 images. In the developing cohort, 374 images are used for training the improved ResNet + SVM model and the traditional ResNet model respectively. In the developing cohort, 352 images are used for training the multi-model. And the rest 184 images are the validation cohort for calculating and comparing the accuracy of these models.

### Model performance evaluation

For the model performance evaluation, since it is a classification model, we referred to Jake Lever et al. [28] which is published in Nature Methods. And in the Artificial intelligence industry, Recall score, precision, F1 score, and ROC curve are the mainstream evaluation indicators for classification problems. In Jake Lever et al., classifiers are commonly evaluated using either a numeric metric, such as precision, or a graphical representation of performance, such as a receiver operating characteristic(ROC) curve. Jake et al. shows that classification metrics are calculated from true positives(TPs), false positives(FPs), false negatives(FNs), and true negatives(TNs), all of which are tabulated in the so-called confusion matrix. A confusion matrix is a table that is often used to describe the performance of a classification model on test data. In the confusion matrix, each column represents the model prediction for each category, and each row means the actual label for each category.

For this binary classification problem, when one instance is predicted by the model, four situations are as follows:

1. If the true label of the instance is “Positive” and is predicted as a “Positive” class by the model, it is a true class, called “True Positive”, and marked as TP;
2. If the true label is “Positive”, while is predicted as “Negative” class by the model, called “False Negative”, and marked as FN;
3. If the true label is “Negative”, while is predicted as a “Positive” class by the model, called “False Positive”, and marked as FP;
4. If the true label is “Negative”, and the model result is also “Negative”, called “True Negative”, and marked as TN.

In this paper, “Positive” means Electron dense granules are present in the TEM image. And “Negative” means Electron dense granules are not present in the TEM image.

#### Recall score

Recall score measures how many “Positive” samples are predicted by the model as “Positive”. The recall score expresses the model's ability to find all positive instances which are EDD present in the TEM in the dataset. Recall score measures whether the model omits the true positive instances. The closer the recall score is to 1, the higher the accuracy of the model prediction. The formula of the recall score is:

4 $$\mathrm{recall}=\frac{TP}{TP+FN}$$

#### Precision

Precision indicates the proportion of “Positive” cases that are divided into “Positive”. Precision shows how much of the model predicted positive was correct. Precision measures the accuracy of the model in determining ‘positive’. The closer the precision is to 1, the higher the model accuracy is.

Precision is calculated as:

5 $$\mathrm{P}=\frac{TP}{TP+FP}$$

#### F1 Score

The precision and recall score sometimes have contradictions, so they need to be considered comprehensively. The most popular classification evaluation is the F1 score, and it is the balance between recall and precision. It can measure the model prediction accuracy comprehensively. The value of the F-score is between 0 and 1. The closer to 1, the higher the model accuracy is.

The formula of F-Score is:

6 $$\mathrm{f}1=\frac{2\bullet P\bullet recall}{P+recall}$$

Considering that the image sample size is very small in this study, it is difficult to improve the model accuracy. As a result, we think that a 5% model accuracy improvement is significant.

#### ROC Curve

ROC curve is short for the receiver operating characteristic curve. Each point on the ROC curve reflects the susceptibility to the same signal stimulus. The horizontal axis of the ROC curve is the specificity of the false positive rate FPR, and the vertical axis is the sensitivity of the true positive rate TPR. AUROC is the area under the ROC curve. It is used to evaluate the model's accuracy. And the closer it is to 1, the higher the model accuracy is. Among them, the formula for calculating FPR and TPR are as follows respectively:

7 $$\mathrm{FPR}=\frac{FP}{FP+TN}$$

8 $$\mathrm{TPR}=\frac{TP}{TP+FN}$$

## Results

### Patient characteristics

In the Children’s Hospital of Nanjing Medical University from June 2014 to June 2019, 1252 patients were required for a renal biopsy. Among them, 319 patients were required TEM checks. Among the 319 patients, in clinical diagnosis, 22 cases were suspected of lupus nephritis, 103 cases of purpura nephritis, 16 cases of IgA nephropathy, and 2 cases of IgM nephropathy. All of these belong to immune-mediated renal disease, and these add up to a total of 143 cases. As a result, there are 143 cases of clinically suspected immune complex nephritis in total that need to be excluded or diagnosed by TEM.

### Feature map

For the TEM image, the feature map results from each layer in the ResNet50 model are shown in Fig. 6. For better visualization, the feature maps are reshaped to 2D pictures. In the training phase, since it is a transfer learning task, the learning rate is set at 0.00001, the optimizer is “Adam”, and the loss is the cross-entropy and 10 epochs.

**Fig. 5:**
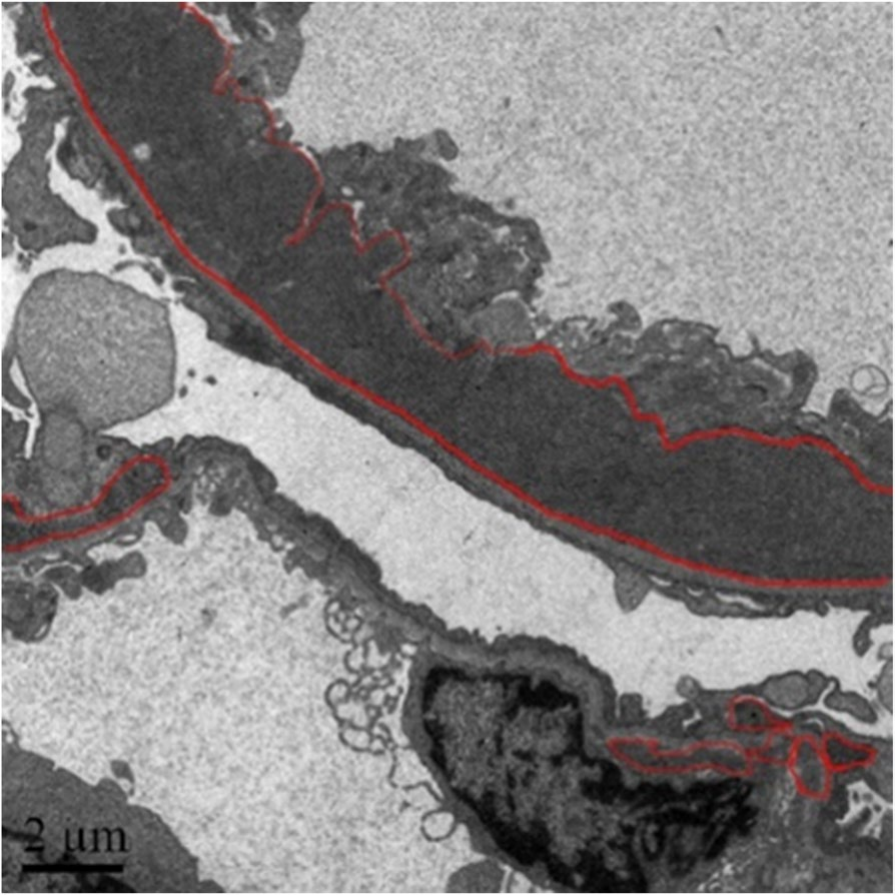
Original TEM Image with “Positive” Lable

Figure 5 shows one original TEM image of a renal biopsy from the patient. This image is labeled as “Positive” which means that the electron-dense granule is present in this image. In this picture, the electron-dense granules are outlined in red on this image. Then Fig. 5 will be fed into the model for feature extraction.

**Fig. 6:**
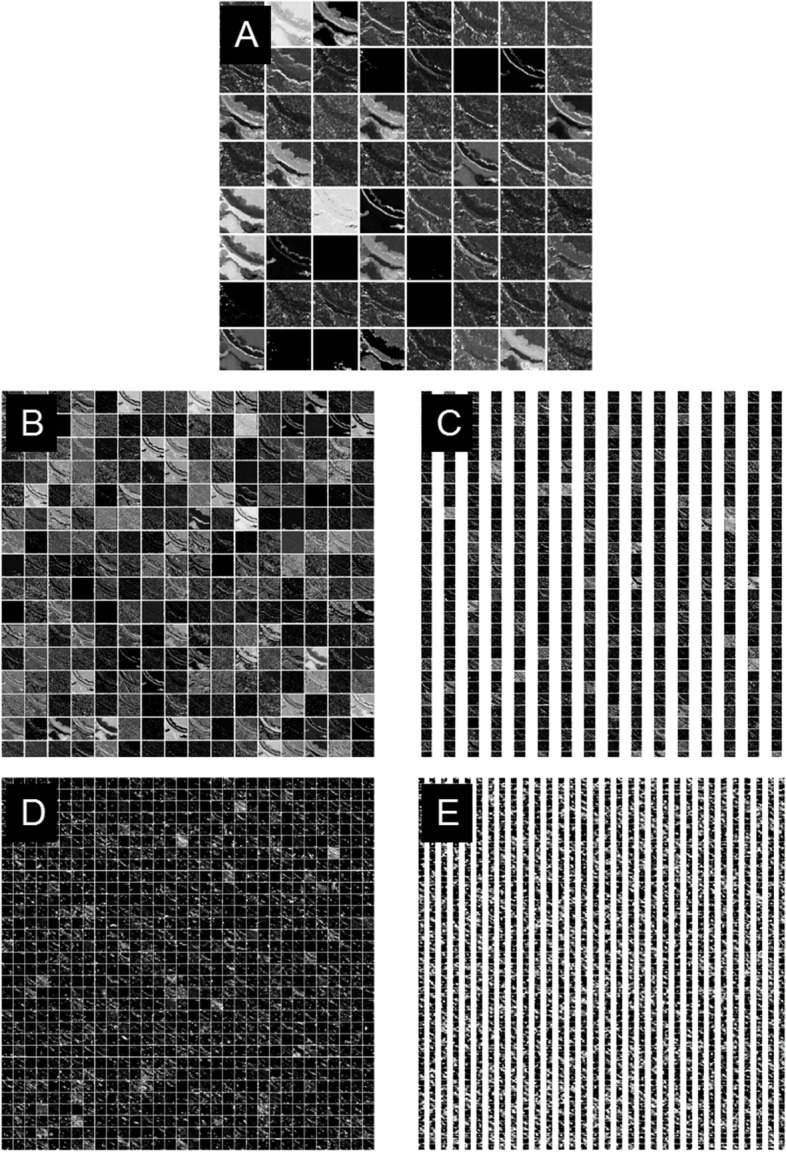
Feature Maps of Deep Learning Model. **A** Extracted feature map from stage1 of deep learning model. **B** Extracted feature map from stage 2 of deep learning model. **C** Extracted feature map from stage3. **D** Extracted feature map from stage4. **E** Extracted feature map from stage5

Figure 6 shows all the feature maps from the improved ReseNet + SVM feature extraction network by sequence. The feature map’s size is 3-dimensional [height, width, channe]. In Fig. 6, the sub-figure marked as “A” is the feature map from the Convolutional layer of stage 1, and its size is [56, 56, 64]. From Fig. 6-A, there are 64 small patches, each patch represents the feature of the original TEM image, and each patch’s size is 56 × 56. The 64 image features are different from each other, and they contain different information from the original image. For better visualization, the 3D feature maps are reshaped to 2D, and the channel is the number of small patches. Figure 6-B, C, D, E show the feature maps from stage 2, stage 3, stage 4 and stage 5 successively, and the sizes are [56, 56, 256], [28, 28, 512], [14, 14, 1024] and [7, 7, 2048]. Figure 6-A corresponds to the “Feature-map 1” marked in Fig. 3, and Fig. 6-B, C, D, and E corresponds to the “Feature-map 2”, “Feature-map 3”, “Feature-map 4”, “Feature-map 5” in Fig. 3 respectively.

From Fig. 6, it’s obvious that the deeper the network, the height and width of a single image patch are smaller, and the channel is larger. Besides, the feature map from the shallow net focuses on more detailed geometry information of the partial area of an image, and the feature map contains fine information. While the feature map from the deeper net, the feature map focuses on more global and coarse information of the image.

### Confusion matrix results

Table 1, Table 2, and Table 3 show the performances of the conventional Resnet50 model, improved Resnet50 + SVM model, and deep learning-based multi-model respectively. In the confusion matrix, “Positive” means Electron dense granules are present in the TEM image. And “Negative” means Electron dense granules are not present in the TEM image.

**Table 1:** Confusion Matrix of Conventional ResNet Model

**Conventional ResNet50 Model** Confusion Matrix		Prediction Result	
		Positive	Negative
Ground Truth	Positive	39	43
	Negative	26	56

The top left corner 39 is the number of “TP”, 43 is the “FN”, 26 is the “FP”, and 56 is the “TN”

**Table 2:** Confusion Matrix of Improved ResNet + SVM Model

**Improved ResNet50 + SVM Model** Confusion Matrix		Prediction Result	
		Positive	Negative
Ground Truth	Positive	65	17
	Negative	10	72

The top left corner 65 is the number of “TP”, 17 is the “FN”, 10 is the “FP”, and 72 is the “TN”

**Table 3:** Confusion Matrix of Deep Learning Based Multi-model

**Deep Learning Based Multi-Model** Confusion Matrix		Prediction Result	
		Positive	Negative
Ground Truth	Positive	70	12
	Negative	6	76

The top left corner 70 is the number of “TP”, 12 is the “FN”, 6 is the “FP”, and 76 is the “TN”

For the “True Positive” (TP) and “False Negative” (FN) values, the deep learning-based multi-model has the largest values which are 70 and 76 respectively. The “TP” and “FN” values of the Improved ResNet50 + SVM model are much larger than the conventional ResNet50 model. What’s more, for the values of “False Positive” and “False Negative”, Multi-model has the best performance, and the improved ResNet50 + SVM model is better than the conventional ResNet50 + SVM. From the confusion matrix of the three models, it’s obvious that the deep learning-based multi-model gets the highest accuracy, and the improved ResNet50 + SVM model is more accurate than the conventional ResNet50 model.

### ROC curve

**Fig. 7:**
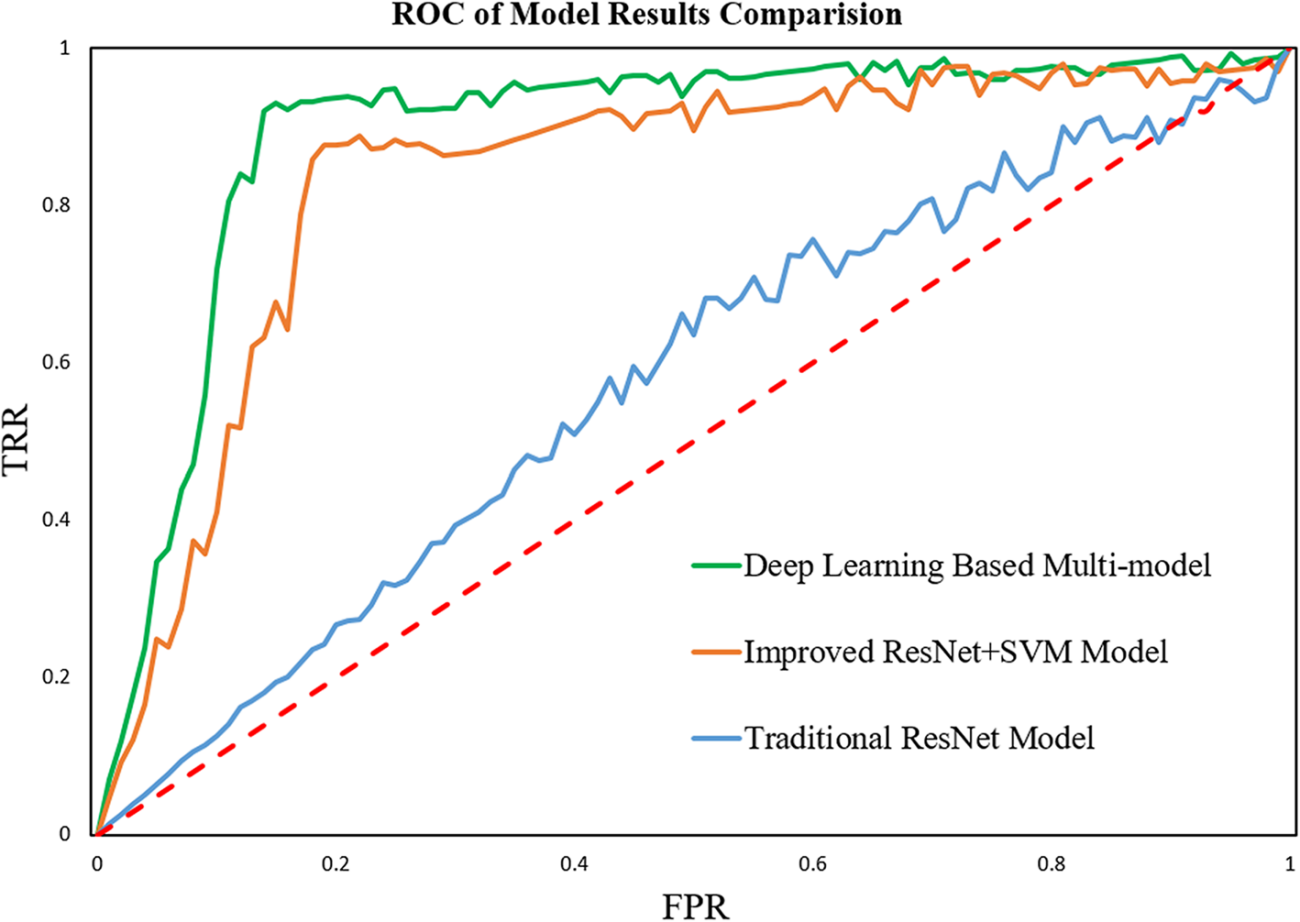
ROC Curve of Model Results Comparison

Figure 7 is the ROC curve of the three models. The horizontal axis is the False Positive Rate, and the vertical axis is the True Positive Rate. The green curve is the ROC curve of the Deep Learning-Based Multi-model. The orange curve is the ROC of the Improved ResNet + SVM model. And the blue curve is the ROC of the traditional ResNet model. The red dashed line is the diagonal line, which corresponds to the “random guessing” model (50% possibility of guessing right). The closer the ROC curve is to the upper left corner, the better the prediction performance of the model. For the point in the upper left corner which is (0, 1), it corresponds to the “ideal model” where the FPR = 0 and TPR = 1, which means that the model classified all the samples correctly. It can be seen from the above figure that the ROC curve of the multi-model is more skewed to the upper left corner, the model accuracy is higher. The ROC curve of the improved ResNet + SVM model performs better than the conventional ResNet model.

The traditional ResNet model AUROC is 0.579. And the improved ResNet50 + SVM model AUROC is 0.835. The deep learning-based multi-model AUROC is 0.890. It shows that deep learning-based multi-model has the highest model accuracy.

### Accuracy

Table 4 shows the precision, recall, and F1 scores of the three models respectively. In Table 4, the improved ResNet50 + SVM model precision and recall scores are 87% and 79% respectively, while the ResNet50 model precision and recall scores are only 60% and 48%. And the proposed improved ResNet50 + SVM model is much more accurate than the traditional ResNet50 model, which improved about 27% accuracy for precision and 31% accuracy for recall score. What’s more, deep learning-based multi-model precision and recall scores are 92% and 85%, which are about 5% more accurate than the improved ResNet50 + SVM single model.

**Table 4:** Precision, Recall and F1 Scores of Three Models

	**ResNet50**	**ResNet50 + SVM**	**Multi-model**
Precision-score	0.60	0.87	0.92
Recall-score	0.48	0.79	0.85
F1-score	0.53	0.83	0.88

In general, the Deep learning-based multi-model first proposed by this paper has the highest model accuracy, the F1 score is about 88%. And Improved ReseNet50 + SVM model also first proposed by this paper performance is much better than the traditional ResNet50 model. And the average accuracy of the improved ResNet50 + SVM model is 83%, while the traditional ResNet50 model accuracy is only 53%.

Although the traditional ResNet50 model is the state-of-the-art image classification model, the improved ResNet50 + SVM and multi-model have much better performance. What’s more, the multi-model performs better than the Improved ResNet + SVM model, which improves about 5% accuracy.

## Discussion

Transmission Electron Microscope (TEM) is performed routinely on renal biopsies.

Especially for immune-mediated renal disease diagnosis, whether the electron-dense granule is present in the electron microscope report is of vital importance.

When the clinical symptoms are highly suspected of immune-mediated nephritis, the renal biopsy specimens can be obtained to feed into the model, and model results can quickly assist the final diagnosis of immune-mediated nephritis. Immune glomerulonephritis is caused by the deposition of immune complexes in the kidney. For example, IgA nephropathy is a common primary immune glomerulonephritis, which is characterized by the deposition of IgA in the glomerular mesangial area. At present, the main pathogenesis of the disease is that galactose deficient IgA1 and its related anti-sugar antibodies form immune complexes in the kidney, which cause IgA nephropathy. IgA nephropathy was judged by the presence of an immune complex in the kidney The presence of an immune complex in the kidney is one of the main pieces of evidence suggestive of IgA nephropathy. Lupus nephritis is one of the common secondary immune nephritis, which is mainly caused by the deposition of autoantibodies in the kidney and the activation of autoimmune response by the combination of immune complex and intrinsic renal antigen. There must be immune complexes in the kidney of immune-mediated glomerulonephritis, so the presence or absence of immune complexes in the electron microscope can assist in the diagnosis of immune glomerulonephritis. If the disease is mild, there may not be immune complex deposition in the kidney at this time. Since substances with a molecular weight of less than 70,000 can flow out with urine through the glomerular filtration membrane, in the case of mild kidney disease, if there is an immune complex with a molecular weight of less than 70,000, it may flow out with urine and cannot be deposited in the kidney. In addition, specimens taken from the renal biopsy are random and may not be able to accurately obtain the lesion site. At this time, the site of immune complex deposition will be missed. There is no immune complex in the electron microscopic specimen of immune nephritis. These are the limitations of electron microscopy. Therefore, the diagnosis of immune complex-mediated nephritis needs to be combined with clinical symptoms, light microscopy, electron microscopy, and so on. This is also the advantage of this algorithm. It can search the whole specimen, find whether the whole specimen has no electronic dense matter, and even can search more than one specimen of the same patient efficiently. Deep learning model training takes a long time. But once the model has been trained, the model inference process is carried out for pathological image identification, which is very fast. It only often takes 1 s. In this experiment, the model inference process takes 1 s. In addition, the computer can continuously diagnose and identify. It is difficult for humans to work continuously for a very long time with no rest. So in general, models will assist pathologists to diagnose quicker. At the same time, the algorithm trained by more data can distinguish the electron-dense matter and the impurity interfering matter which is similar to the electron-dense matter better than the human eye.

And the immune complex renal disease can be judged by the presentence of electron-dense granules. Electron-dense granule helps to support the diagnosis of immune-mediated nephritis, and if the electron-dense granule is present, it is helpful to support the clinical suspect of immune-mediated nephritis. As a result, the existence of an electron-dense granule is crucial. For better TEM medical image classification accuracy and efficiency, this study improved the conventional image classification deep learning models and developed a novel method of deep learning-based multi-model.

More recently, there has been a growing number of publications focusing on deep learning applications for disease diagnosis. Although some papers describing the deep learning models on renal biopsy are published recently, their tasks are about colorful images such as PAS sample images. Previous studies of renal biopsy have not dealt with deep learning models on TEM images. Since the TEM image is grayscale which contains less pixel information compared with colorful images, the morphological changes in greyscale image (TEM) are much harder to identify for the computer. What’s more, most machine learning methods on renal disease are used for segmentation problems, which is a pixel-level classification problems. While in our study, it is a whole image classification problem, which is image-level classification.

Since TEM images require needling biopsy of the renal, which is a traumatic examination and has certain risks, the images are scarce. Especially for deep learning model training, hundreds of images are too small. Although in this study transfer learning is adopted to alleviate the problem of lack of data, the small amount of data is still the main reason for the low accuracy of the deep learning models.

These methods have practical value in real life. For the regions which are rich in medical resources with experienced pathologists, it could assist the pathologist. And for the developing regions which lack good medical resources and experienced pathologists, deep learning models could be helpful for the development of renal biopsy.

What’s more, the images are from patients who are children. The model performance on adult TEM images can be explored. This research only focuses on the TEM result, in the further study, clinical symptoms, light microscopy, and immunofluorescence results will be integrated to assist in the clinical diagnosis of immune complex-mediated nephritis. Our research serves as a pioneer and reference for further research on renal biopsy. In further study, semantic segmentation models can be developed to detect the location, pattern, and extent of electron-dense granules. Future research to make artificial intelligence fully applicable to all renal biopsy images is encouraged.

**Figure:**
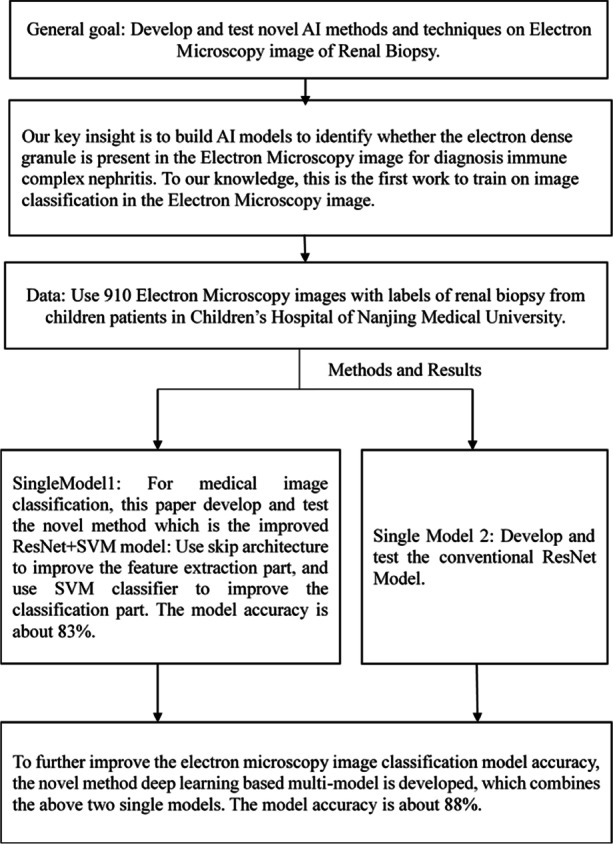
A simple schematic drawing illustrating the main concept of the work

## Data Availability

The data and materials are not available. Because Children’s Hospital of Nanjing Medical University has the data rights, and Children’s Hospital of Nanjing Medical University cherishes the data and chooses not to disclose the data for the time being. If the editors think there is a need to disclose the data, we can apply to the Children’s Hospital of Nanjing Medical University again.
